# Generator and Lead-Related Complications of Implantable Cardioverter Defibrillators

**Published:** 2014-04-01

**Authors:** Ahmad Yaminisharif, Nader Soofizadeh, Akbar Shafiee, Ali Kazemisaeid, Arash Jalali, Ali Vasheghani-Farahani

**Affiliations:** 1 Tehran Heart Center, Tehran University of Medical Sciences, Tehran, IR Iran

**Keywords:** Implantable Cardioverter Defibrillators, Complication, Implanted Electrodes

## Abstract

**Background:**

Increase in the number of patients treated with Implantable Cardioverter Defibrillator (ICD) requests more attention regarding its complications.

**Objectives:**

This study aimed to assess the generator- and lead-related complications at implantation and during follow-up in the patients who were treated with ICD for primary and secondary prevention reasons.

**Methods:**

We retrospectively reviewed 255 consecutive patients who underwent transvenous ICD implantation for the first time in a 7-year period and were followed-up for 3 years at Tehran Heart Center. The personal and clinical data of the patients as well as specific data on the ICD implantation were retrieved. The frequency of each of the complications was reported and the study variables were compared between the patients with and without complications using Student’s t-test and chi-square test where appropriate. P values less than 0.05 were considered as statistically significant.

**Results:**

Out of a total of 525 implanted leads and 255 implanted devices in 255 patients (mean age = 62.57 ± 13.50 years; male = 196 [76.9%]), complications leading to generator or lead replacement occurred in 32 patients (12.5%). The results revealed no significant difference between the patients with and without complications regarding gender and age (P = 0.206 and P = 0.824, respectively). Also, no significant difference was found between the two groups concerning the ejection fraction (P = 0.271). Lead fracture was the most frequent lead-related complication and was observed in 17 patients (6.6%). Besides, it was mainly observed in the RV leads. Generator-related complications leading to generator replacement were observed in 2 patients (0.7%).

**Conclusions:**

Despite considerable improvements in the ICD technology, the rate of the ICD complications leading to device replacement and surgical revision, especially those related to the leads, is still clinically important.

## 1. Background

The use of Implantable Cardioverter Defibrillator (ICD) in prevention of sudden cardiac death has been increased dramatically in the past few decades (1). The patients with structural heart disease and symptomatic sustained Ventricular Tachycardia (VT) or ventricular fibrillation are the prime candidates (2). Recent years have witnessed a dramatic rise in the number of patients receiving treatment with ICD and other cardiac devices and, thereby, complications necessitate timely identification and treatment (3). Currently, about 30% of the patients who receive an ICD experience at least one complication after the implantation (4). A study on the frequency and incremental cost of major complications among the patients with an ICD showed that 10.8% of the patients experienced one or more complications, and this was accompanied by a significant increase in the length of hospital stay and health service costs (5). Device related complications can be classified into generator- and lead-related complications. ICD lead failure is frequent and mostly stems from insulation defect or conductor disruption, and can affect the high-voltage or the pace-sense circuit of the lead (6, 7). The most common complications of lead failure in previous studies included oversensing electrical noise, undersensing ventricular tachyarrhythmias, inappropriate therapy, and lethal proarrhythmia (8, 9).

Up to now, several studies have been conducted on the ICD-related complications, such as inappropriate shocks, infection, and endocarditis. However, data on the complications directly related to the ICD leads or generator malfunction are limited.

## 2. Objectives

Hence, the present study aims to assess both the generator-related and lead-related complications at implantation and during follow-up in the patients who were treated with an ICD for primary and secondary prevention reasons at Tehran Heart Center, Tehran, Iran.

## 3. Patients and Methods

### 3.1. Patients

In this study, clinical data of 255 patients who underwent first-time transvenous ICD implantation between March 2003 and February 2009 at Tehran Heart Center and had at least 3 complete follow-up visits within 3 years following device implantation were retrospectively reviewed. General characteristics of the study population and its clinical data as well as specific data on the ICD implantation were recorded for each patient. All the patients signed a written informed consent in accordance with the protocols of Tehran Heart Center at the time of admission, granting approval for the anonymous use of their data in medical research. Also, the proposal of the present study was approved by the Research Board of Tehran Heart Center.

### 3.2. ICD Implantation and Follow-up

Indications for the ICD implantation were based on the contemporary guidelines and consisted of primary and secondary prevention (10). The implantation procedure was performed in the Electrophysiology Laboratory of Tehran Heart Center by five electrophysiologists who were experienced in device therapy. All the patients received multi-programmable ICDs with pacing capabilities and automatic intracardiac electrogram storage for the events triggering device therapy. The ICD system manufacturers included Medtronic, St. Jude, and Bostone Scientific. Lead implantation was performed via the transvenous approach through subclavian or axillary access using non-thoracotomy lead systems. In case of failure in transvenous insertion, the Left Ventricular (LV) lead was placed in the epicardial position in the operating room. The efficacy of pacing, sensing, and defibrillation was assessed after positioning the ICD lead. Moreover, defibrillation testing was performed using 10 - 15 joules of energy initially. If the arrhythmia failed to respond in the first step, the second step was to utilize 20 - 25 joule shocks and then with maximum energy as the last resort. The ICD evaluations during the follow-up visits involved routine clinically appropriate measurements, including interrogation of the device for tachyarrhythmia episodes, evaluation of sensing and pacing thresholds, lead impedance, and battery status. Data on the ICD-related complications were reviewed from the patients’ records.

### 3.3. Classification of Complications

In this study, the ICD-related complications were divided into two main categories: the complications related to the generator and those resulting from lead problems. The generator-related complication was generator malfunction. On the other hand, the lead-related complications included the following situations: dislodgement defined as X-ray-confirmed dislodgement of the lead combined with significant changes in sensing / pacing performance, failure to capture at practical device output with no visible change in the lead position or considerable impedance rise, loose set screw at the ICD connector, lead insulation defect, and lead fracture defined as changes in impedance with changes in sensing / pacing performance (intermittent or permanent) which could be optionally confirmed by X-ray study. Suboptimal and abnormal findings in the device analysis were defined as threshold > 2 V, Right Atrial (RA) lead impedance > 1500 Ω, Right Ventricular (RV) lead impedance > 2000 Ω, LV lead impedance > 1500 Ω, RV coil > 200 Ω, SVC coil > 200 Ω, R-wave < 5 mV, and p-wave < 0.5 mV.

### 3.4. Statistical Analysis

The statistical analyses were performed using the PASW 18.0 software package for Windows (SPSS, Inc., Chicago, IL, USA). The continuous variables were expressed as mean ± 1 Standard Deviation (SD) and were compared using Student’s t-test. Additionally, comparison of the dichotomous categorical variables and comparisons between the patients with and without complications were made using chi-square test. P values less than 0.05 were considered as statistically significant.

## 4. Results

In this study, the follow-up data of 255 consecutive patients (mean age = 62.57 ± 13.50 years; male = 196 [76.9%]) who underwent ICD implantation at Tehran Heart Center were reviewed. General characteristics of the patients have been summarized in Table 1. The indication of device implantation in almost half of the patients (54.1%) was secondary prevention. There were 8 patients (3.1%) in this study who died during the follow-up period. The characteristics of the implantable devices, including their type and manufacturer, are depicted in Table 2. 

**Table 1.** General Characteristics of the Study Population ^[Table-fn fn8152]^

Variable	Study Population (n = 255)
**Age, year (mean ± SD)**	62.57 ± 13.50
**Male gender, n (%)**	196 (76.9)
**CAD, n (%)**	173 (67.8)
**Diabetes mellitus, n (%)**	70 (27.5)
**Hypertension, n (%)**	83 (32.5)
**Hyperlipidemia, n (%)**	75 (29.4)
**Smoking, n (%)**	58 (22.7)
**CRF, n (%)**	90 (35.3)
**EF, % (mean ± SD)**	29.55 ± 11.91
**EF < 35%, n (%)**	169 (66.2)

Abbreviations: CAD, Coronary artery disease; CRF, Chronic renal failure; EF, Ejection fraction; SD, Standard deviation

**Table 2.** Characteristics of the ICDs in the Study Population ^[Table-fn fn8153]^

Variable	Study Population (n = 255)
**ICD type, n (%)**	
Single chamber device	65 (25.4)
Dual chamber device	110 (43.1)
Biventricular device	80 (31.3)
**ICD manufacturer, n (%)**	
Medtronic	140 (54.9)
St. Jude	109 (42.7)
Bostone	6 (2.4)
**RA leads (n = 190)**	
Capsure	110 (57.8)
Tendril	66 (34.7)
Isoflex	11 (5.7)
Flextend	3 (1.5)
**RV leads (n = 255)**	
Endotalk	2 (0.7)
Sprint Quattero	101 (39.6)
Riata	79 (30.9)
Durata	32 (12.5)
Sprint Fidelis	41 (16.0)
**LV leads (n=80)**	
Attain	47 (58.7)
QuickSite	24 (30.0)
Epicardial	5 (6.2)
QuickFlex	4 (5.0)

Abbreviations: ICD, Implantable cardioverter defibrillator; LV, Left ventricle; RA, Right atrium; RV, Right ventricle

Out of a total of 525 implanted leads and 255 implanted devices, complications leading to generator or lead replacement occurred in 32 patients (12.5%). The results showed no significant difference between the patients with and without complications regarding gender and age (P = 0.206 and P = 0.824, respectively). Also, no significant difference was found between the two groups concerning the ejection fraction (P = 0.271).

During the follow-up period, generator-related complications leading to generator replacement were observed in 2 patients (0.7%). One case was due to high-voltage circuit damage and the other one had generator software corruption following Magnetic Resonance Imaging (MRI). It should be noted that the type of the generator was not associated with the complications. 

Lead-related complications included skin erosion due to lead, lead displacements; lead fractures, insulation defects, and loose set screw (Table 3). Among these complications, lead fracture was the most frequent one and was observed in 17 patients (6.6%). Besides, it was mainly observed in the RV leads. Lead fracture was more frequently observed with Sprint Fidelis® leads; albeit, it was not statistically significant (P = 0.287) (7 leads out of 40 Sprint Fidelis® leads were complicated). Riata® leads were the second most fractured leads with an overall number of 5 fractures. Only 30 patients (11.7%) underwent lead reimplantation and the rest were treated by changing the device mode or omitting the lead from the circuit. No cardiac perforation was recorded in the study population. Details of the findings in the device analysis are described in Table 4. 

**Table 3.** Lead-Related Complications ^[Table-fn fn8154]^

Complication	N (%)
**RA lead (n = 190)**	
Skin erosion due to lead	2 (1.0)
Lead fracture	2 (1.0)
Displacement	3 (1.5)
Total	7 (3.6)
**RV lead (n = 255)**	
Skin erosion due to lead	2 (0.7)
Lead fracture	14 (5.4)
Displacement	4 (1.5)
Insulation break	1 (0.3)
Decreased R-wave amplitude	1 (0.3)
Total	22 (8.6)
**LV lead (n = 80)**	
Loose set screw	1 (1.2)
Lead fracture	1 (1.2)
Displacement	1 (1.2)
Total	3 (3.7)

Abbreviations: LV, Left ventricle; RA, Right atrium; RV, Right ventricle

**Table 4.** Suboptimal and Abnormal Findings in the Device Analysis (Abnormal Findings Are Marked with Asterix) ^[Table-fn fn8155]^

Parameters	N (%)
**RA-lead related (n = 190)**	
RA threshold > 2 V	5 (2.6)
RA impedance > 1500 Ω	2 (1.0)
P-wave < 0.5 mV	7 (3.6)
**RV-lead related a (n = 255)**	
RV threshold > 2 V	20 (7.8)
RV impedance >2000 Ω	10 (3.9)
R-wave < 3 mV	29 (11.3)
RV coil > 200 Ω	2 (0.7)
SVC coil > 200 Ω	2 (0.7)
**LV-lead related (n = 80)**	
LV threshold > 2 V	19 (23.7)
LV impedance > 2000 Ω	4 (5.0)

Abbreviations: LV, Left ventricle; RA, Right atrium; RV, Right ventricle; SVC, Superior vena cava

## 5. Discussion

This study showed that lead- or generator- related complications in almost 12.5% of an unselected population of the patients receiving ICD resulted in lead and device replacement. The risk of these complications was not influenced by any patient-specific factor, such as age and gender, or device-specific factors. Our findings are in line with those of other studies that have reported an almost similar risk of complications following ICD implantation (4).

Despite the growing number of the ICD implantations, modern ICDs have fewer complications compared to the first generations (11). Previous studies have reported a wide variety of complications after ICD implantation. They were not, however, consistent with respect to the rate of the events related to the lead or the generator.

The rate of generator-related complications in the present study was very low. It should be noted that we only described technical issues and other complications leading to device replacement, such as infection, were excluded from our analysis. The current evidence shows that the risk of generator-related complications is higher for ICD than that for the pacemaker (12). Prolonged charging periods and early battery depletion are the most frequent reported complications of the ICD generator, while prophylactic replacement after the manufacturer recall accounts for the majority of the cases with ICD replacement (4, 13, 14). The limited number of generator-related complications in the present study precluded the identification of any specific risk factor.

In the present study, most of the device replacement procedures were performed due to lead problems, which is in line with the previous studies (15, 16). Moreover, the principal reason for lead replacement was microfracture. However, it has been reported previously that lead failures mostly involved dislodgements (15-17). RV lead complications were observed more frequently and, therefore, comprised more cases of device replacement. This finding is consistent with those of other studies that discussed lead problems and may be related to the higher number of RV leads in this study (15-17). The prevalence of lead complications may imply the fact that leads are more susceptible to damage and injury and special consideration is required during lead insertion and fixation. Although age and gender have been reported as risk factors for defibrillation lead defects, their influence was not significant in the current study (18, 19). It should be noted that not every patient with abnormal or suboptimal analysis needed device reimplantation. Therefore, based on the electrophysiologist’s opinion, some patients were treated by reprogramming the device or omitting the malfunctioning lead.

Manufacturer and technical parameters of the leads have been shown to influence their function and durability (17, 20, 21). In this study, a higher rate of lead fracture was observed with the Sprint Fidelis® leads; albeit, it was not statistically significant (7 out of 17 cases, 41.1%). Sprint Fidelis® is a family of small-diameter leads released by Medtronic (Minneapolis, MN, USA) in 2004 with a 6.6 Fr lead body. In 2007, Medtronic Inc. announced withdrawal of the Sprint Fidelis® leads from the market due to malfunction and performance below the company’s standards (22). Since that time, studies have shown a higher rate of complications with Sprint Fidelis® leads (23-25). Despite these observations, the current data suggest that the decision to perform prophylactic lead replacement must include a weighing of the potential risks of lead revision against the risks of lead failure (25).

Our study provided a good overview of the generator and lead problems in the patients who received ICD within a 5-year period. Nevertheless, the study had a few limitations. First, we only included the patients at Tehran Heart Center and, consequently, our results may not be generalizable. Second, there is a minimal probability that a small number of the patients had also been admitted in other centers due to complications and their records were not available for this analysis. Finally, we only included hardware-related complications of the leads and generators; thus, complications, such as infection, were not within the scope of this work.

The rate of the ICD complications leading to device replacement and surgical revision is still clinically important in spite of the recent remarkable improvements in ICD technology. A large number of our patients had suboptimal function in the device interrogation and are, thus, probably prone to device failure in future. These findings are consistent with the previously reported rates of generator- and lead-complications. Further data on risk factors for generator- and lead-related complications and longer follow-up studies will be helpful in recognizing device complications with specific focus on the manufacturers, modifying the treatment guidelines, and improving the device technology.
